# Macular Sensitivity Endpoints in Geographic Atrophy: Exploratory Analysis of Chroma and Spectri Clinical Trials

**DOI:** 10.1016/j.xops.2023.100351

**Published:** 2023-06-12

**Authors:** Dolly S. Chang, Natalia F. Callaway, Verena Steffen, Karl Csaky, Robyn H. Guymer, David G. Birch, Praveen J. Patel, Michael Ip, Simon S. Gao, Jayla Briggs, Lee Honigberg, Phillip Lai, Daniela Ferrara, Yasir J. Sepah

**Affiliations:** 1 Genentech, Inc., South San Francisco, California; 2 Byers Eye Institute, Stanford University School of Medicine, Palo Alto, California; 3 Retina Foundation of the Southwest, Dallas, Texas; 4 Centre for Eye Research Australia, Royal Victorian Eye and Ear Hospital, University of Melbourne, Melbourne, Australia; 5 NIHR Biomedical Research Centre at Moorfields Eye Hospital NHS Foundation Trust and UCL Institute of Ophthalmology, London, United Kingdom; 6 Doheny Eye Institute, University of California, Los Angeles, California

**Keywords:** Mesopic microperimetry, Geographic atrophy, Perilesional sensitivity, Responding sensitivity, Visual function

## Abstract

**Purpose:**

To assess different microperimetry (MP) macular sensitivity outcome measures capturing functional deterioration in eyes with geographic atrophy (GA) secondary to age-related macular degeneration (AMD).

**Design:**

Patients were included from 2 identically designed, phase III, double-masked, randomized controlled clinical trials, Chroma (NCT02247479) and Spectri (NCT02247531).

**Participants:**

Patients enrolled were aged ≥ 50 years with bilateral GA and no evidence of previous or active neovascular AMD.

**Methods:**

Patients were randomized 2:1:2:1 to receive through 96 weeks intravitreal lampalizumab 10 mg every 4 weeks (LQ4W), every 6 weeks (LQ6W), or corresponding sham procedures. For this study, mesopic macular sensitivity of the central 20° was assessed using MP-1 microperimeter at selected sites.

**Main Outcome Measures:**

Two exploratory endpoints were developed, namely perilesional sensitivity (average of points adjacent to absolute scotomatous points) and responding sensitivity (average of all nonscotomatous points; > 0 dB at baseline) by using customized masks for each patient. These were compared with conventional MP endpoints (mean macular sensitivity and number of absolute scotomatous points).

**Results:**

Of 1881 Chroma and Spectri participants, 277 agreed to participate in the present study. Of these, 197 (LQ4W, n = 63; LQ6W, n = 68; pooled sham, n = 66) had reliable MP results. Enlargement of GA lesion area by approximately 2 mm^2^/year across treatment groups was accompanied by deterioration in all MP parameters. There was no difference in worsening of macular sensitivity or absolute scotomatous points among treatment groups. Perilesional and responding sensitivities showed greater decline over time than mean macular sensitivity. Change in GA lesion area at week 48 showed better correlation with perilesional sensitivity (r = −0.17) and responding sensitivity (r = −0.20) than mean macular sensitivity (r = −0.03), while the correlation was highest with the number of absolute scotomatous points (r = 0.37).

**Conclusions:**

Perilesional or responding macular sensitivity measured by MP should be considered more sensitive endpoints than mean macular sensitivity for monitoring functional decline over time in GA. Although perilesional, responding, and mean macular sensitivity had weak correlation with GA lesion area, the number of absolute scotomatous points may provide additional information on the anatomic/functional correlation.

**Financial Disclosure(s):**

Proprietary or commercial disclosure may be found after the references.

Geographic atrophy (GA) is an advanced form of age-related macular degeneration (AMD) and is characterized by progressive degeneration of the outer retina, retinal pigment epithelium, and choriocapillaris.^1^^,^^2^ It is the leading cause of irreversible vision loss in those aged ≥ 50 years.^3^ Currently, there are no approved treatments to prevent GA^4^; however, pegcetacoplan has recently been approved to slow progression once atrophy has occurred^5^ and there are promising therapeutics at various stages of clinical development.^1^

Interventional GA clinical trials are commonly designed to measure and evaluate anatomic endpoints that have a strong correlation with disease progression. The most widely used primary endpoint is change in GA lesion area, which relies on evaluation of imaging features anchored on fundus autofluorescence (FAF) as the primary imaging modality.^6^ To date, anatomic changes as assessed by OCT have not been used as primary endpoints for registrational studies when analyzing GA progression. However, OCT allows 3-dimensional optical reconstruction of the retinal tissue with high-definition documentation, which offers a unique opportunity to explore disease pathogenesis and to establish other image biomarkers in earlier disease stages. Various expert groups, such as the Classification of Atrophy Meeting, are endeavoring to define certain anatomic features on OCT that correlate with progression to GA and as such could be potentially validated and used as clinical trial endpoints.^7^

Functional studies that correlate with anatomic changes over time are necessary because visual function is of key importance to patients and physicians. Best-corrected visual acuity (BCVA), which is the traditional functional endpoint in ophthalmology clinical trials, does not have a strong correlation with GA progression.^8^ This is because significant changes in BCVA scores only become apparent when the foveal center is affected. In addition, BCVA does not entirely capture meaningful functional visual decline throughout the macula and does not show a good correlation with patient’s symptoms.^6^ Therefore, although the measurement of BCVA is important, it may not capture the full visual dysfunction of a patient with GA.^6^

Mesopic microperimetry (MP) is a noninvasive psychophysical test that differs from traditional visual function tests, measuring mesopic macular sensitivity with focal light stimulation and using eye tracking technology.^9^ This allows coregistration of the retinal sensitivity map on the fundus image so that repeat testing of the same region can be achieved. Microperimetry testing involves examining specific and discrete areas of the fundus rather than a global functional response.^10^ This enables the investigation of anatomic or functional correlations in GA and allows measurement of a different aspect of retinal function that may be more clinically relevant than BCVA alone in patients with GA. Moreover, MP has high test/retest reliability in patients with early and intermediate stages of macular diseases, when BCVA is usually less affected and good fixation is still possible.^11^

Currently in clinical research, the 2 most commonly evaluated MP endpoints are mean macular sensitivity and number of absolute scotomatous points (i.e., areas of zero sensitivity at the maximum stimulus of light). However, a limitation of using mean macular sensitivity as an outcome measure in GA clinical trials is that it is averaged across all tested points (both functioning retinal points and scotomatous points); thus, the magnitude of change is blunted by scotomatous points and there is loss of spatial information.^12^ Meleth et al. evaluated macular sensitivity during GA progression using MP over a 24-month period and found that perilesional sensitivity (defined as the average sensitivity of responding points immediately adjacent to absolute scotomatous points) and responding sensitivity (defined as the average sensitivity of all nonscotomatous points) decreased significantly over time.^13^ The authors proposed that these MP outcome measures should be further evaluated as potential functional endpoints in future GA clinical trials.^13^

Chroma (NCT02247479) and Spectri (NCT02247531) were 2 phase III trials evaluating the efficacy and safety of lampalizumab in patients with GA secondary to AMD. The primary efficacy outcome of these trials was mean change in GA lesion area from baseline to week 48, which was not met. Microperimetry was an exploratory endpoint to assess functional decline over time.^14^ In a subanalysis of Chroma and Spectri, all visual function outcomes, including BCVA, low-luminance visual acuity, reading speed, patient-reported outcomes, and conventional MP endpoints, i.e., mean macular sensitivity and absolute scotomatous points, deteriorated over time; however, only the MP endpoints were found to correlate moderately with change in GA lesion area while the other endpoints showed only weak correlation.^15^

In the present study, we investigated the 2 exploratory MP endpoints of perilesional sensitivity and responding sensitivity in the detection of functional deterioration caused by GA secondary to AMD. In addition, we aimed to compare these exploratory MP endpoints with the conventional MP endpoints.

## Methods

### Patients

Patients from the Chroma and Spectri trials (NCT02247479 and NCT02247531, respectively) were included in this study. A detailed description of the trial designs has been published previously.^14^ Both trials adhered to the tenets of the Declaration of Helsinki.^14^ Briefly, Chroma and Spectri were two identically designed, phase III, double-masked, sham-controlled trials conducted at 275 sites in 23 countries. Nine-hundred and six patients were enrolled in the Chroma trial and 975 patients were enrolled in the Spectri trial. Patients were randomized 2:1:2:1 to receive, through 96 weeks, to receive 10 mg of lampalizumab every 4 weeks, sham procedure every 4 weeks, 10 mg of lampalizumab every 6 weeks, or sham procedure every 6 weeks. Microperimetry was performed only at selected sites in patients who agreed to take part in the present study. All sites received approval by their institutional review boards or ethics committees. An independent data monitoring committee provided ongoing oversight. Written informed consent was provided by the participants.

### Microperimetric Assessment

Mesopic macular sensitivity was assessed by MP at baseline and weeks 24, 48, 72, and 96 using the MP-1 microperimeter (NAVIS software version 1.7.1; Nidek). Mean macular sensitivity was measured in the presence of a background luminance of 4 apostlibs (1.27 cd/m^2^) using a 68-loci circular grid centered at the preferred retinal locus, covering the central 20° of the macula (10-2 program). The stimulus was Goldmann size III (0.43° diameter) displayed for duration of 200 ms with 1.5-second intervals. A 4-2 staircase strategy was used, with testing intensities ranging from 127 to 1.27 cd/m^2^, which correspond to macular sensitivities of 0 to 20 dB. Patients were allowed 3 attempts to meet the MP screening test criteria. This involved the ability of the study eye to complete the 10-2 68-point exam in ≤ 30 minutes with a reliability ratio ≥ 80% (< 20% false-positive responses).

In the present study, additional quality control was performed based on the following criteria: the grid pattern must be correctly placed with the center on the fovea, the blind spot must be correctly placed on the optical disc, and the follow-up function must be used for follow-up visits. Only if all these criteria were met was the quality of the data deemed adequate to be included in this study.

**Figure 1:**
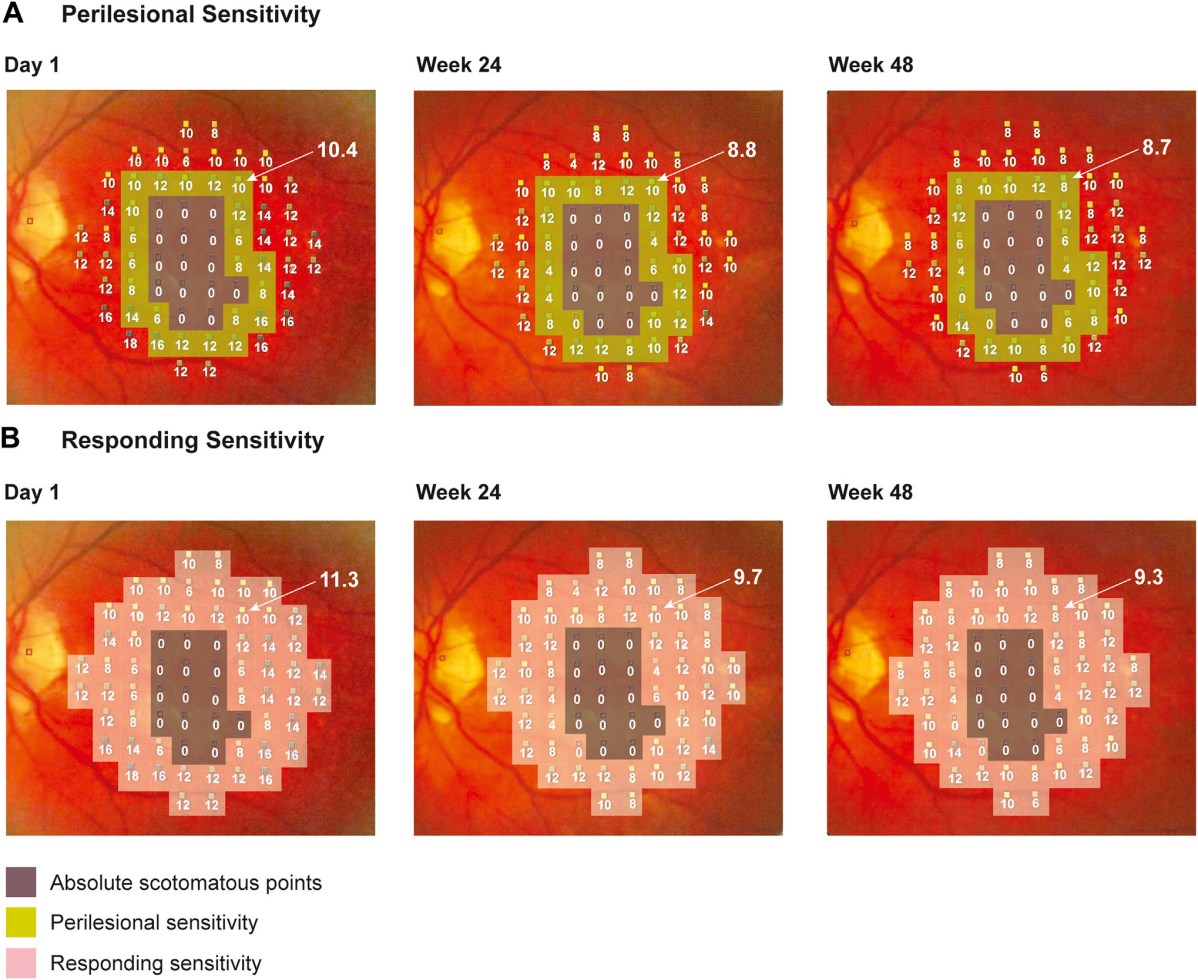
Absolute scotomous points at the baseline visit were located. Subsequently, 2 additional areas of interest were identified that excluded the area of absolute scotomatous points, thereby defining the novel microperimetry endpoints: (**A**) perilesional area that encompasses the rim around the geographic atrophy lesion border, and (**B**) responding area. Perilesional and responding areas were defined at baseline and kept constant over time for any individual eye, even if values in that area became nonresponsive during the study. The values in that area were averaged at each visit to look for changes in perilesional or responding sensitivity over time.

Microperimetry outcome measures that were assessed in this analysis included mean macular sensitivity, perilesional sensitivity, responding sensitivity, and number of absolute scotomatous points.^13^ Mean macular sensitivity was defined as the average of all 68 loci on the circular test grid covering the macula, regardless of their response (including 0 dB). Perilesional sensitivity was defined as the average sensitivity of responding points (MP result > 0 dB at baseline) immediately adjacent to absolute scotomatous points (within all adjacent responding points, including diagonally adjacent points; Fig 1). Responding sensitivity was defined as the average sensitivity of all nonscotomatous points (i.e., > 0 dB at baseline). Absolute scotomatous points were defined as MP testing loci that resulted in no patient response (0 dB; i.e., not seen, even at the maximum stimulus of light) at the baseline visit. In our analysis the 2 types of perilesional and responding sensitivities as well as absolute scotomatous points (filled and 0 points) were combined as both types represent similar clinical visual impairment.

Microperimetry data were imported into MATLAB (MathWorks), and the 68 loci grid was recreated as a matrix. Masks that identify each locus as belonging to a macular region according to these definitions were then generated on the baseline data (Fig 1). The average sensitivity of each of these regions was calculated according to the baseline mask for all visits. Baseline was defined as screening visit or day 1, with a preference for day 1 if MP tests at both visits passed the quality control processes. Longitudinal changes of different MP outcomes determined by interpolation between time points were measured planimetrically.

### Measurement of GA Lesion Area over Time

Color fundus photographs, FAF, and spectral-domain OCT images were collected every 6 months for up to 24 months. Mean change in GA lesion area from baseline was measured by FAF and data were evaluated at the Doheny Image Reading Center (Los Angeles, CA).^14^ Expert graders used RegionFinder (Heidelberg Engineering) to identify the decreased autofluorescence areas on blue-light FAF images and coregistered infrared reflectance images to further guide their processes.^14^

### Statistical Analysis

Mixed-effect model for repeated measure analyses showed no differences for any endpoint between treatment groups when adjusted for baseline GA lesion, subfoveal area, multifocal configuration, biomarkers, BCVA, and sex^14^; therefore, all clinical trial arms were combined for the present analyses. Patients with ≥ 1 follow-up visit were included in the study. Unadjusted means and standard errors were calculated. The variability of the MP outcomes within a time point was assessed using the coefficient of variation (CV; expressed as a percentage), defined as the ratio of the standard deviation to the mean. The CV represented the extent of variability in relation to the mean of the measured characteristics. It was used to evaluate the between-patient variability of each macular sensitivity endpoint at each time point. A lower variability is preferred to detect differences between treatment arms. The correlation between changes from baseline in anatomic features and the functional MP measures at week 48 was assessed using Spearman’s rho, a nonparametric correlation coefficient. All analyses were conducted using R analytics.^16^

## Results

### Baseline Characteristics

**Figure 2:**
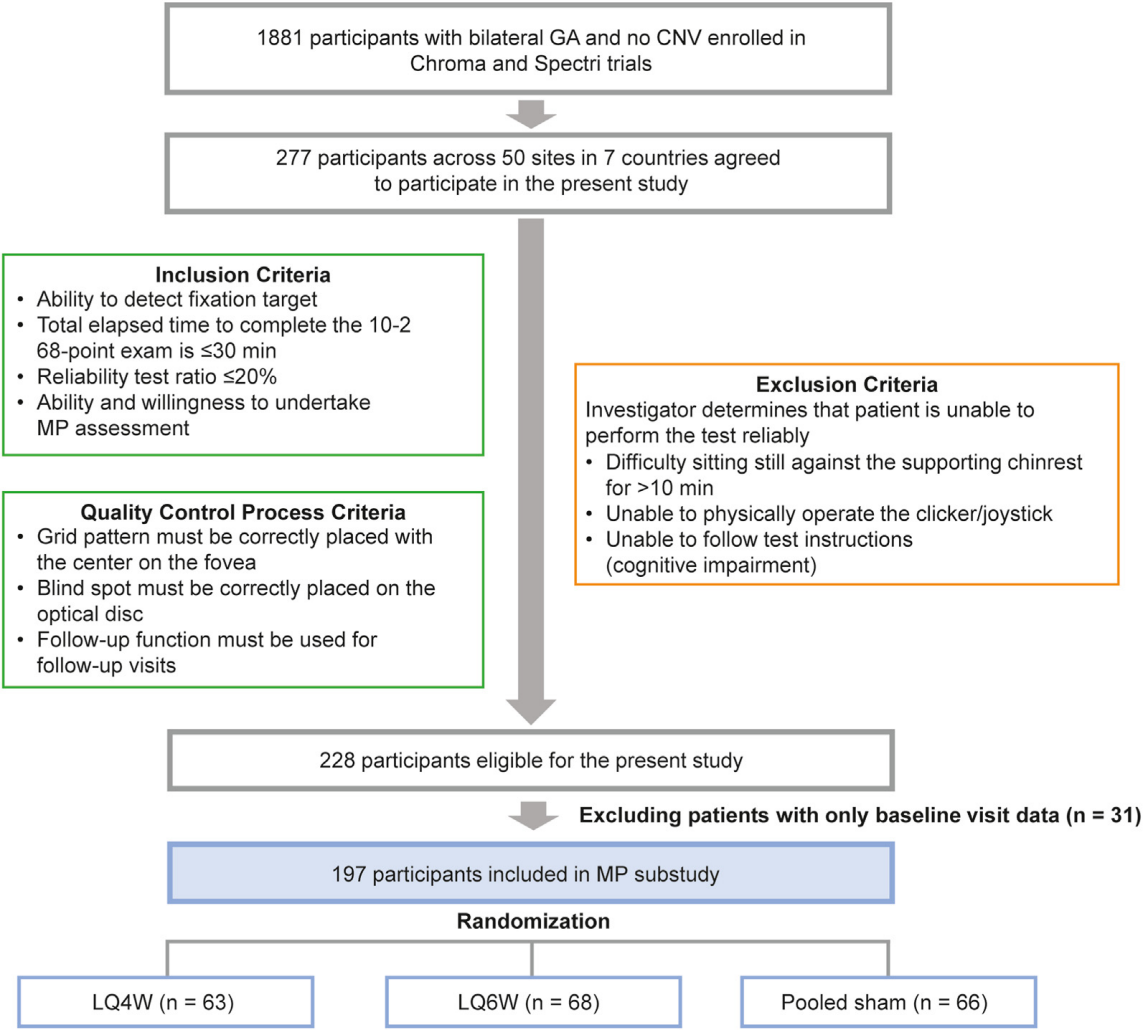
Flow of participants from the Chroma and Spectri lampalizumab phase III trials to the present study. CNV = choroidal neovascularization; GA = geographic atrophy; LQ4W = lampalizumab every 4 weeks; LQ6W = lampalizumab every 6 weeks; MP = microperimetry.

Of the 1881 participants from the Chroma and Spectri trials, 277 (14.7%) across 50 sites in 7 countries agreed to participate in the present study (Fig 2). After the additional quality control process, data from 228 (82.3%) participants were deemed to be of adequate quality to be included in the present study; 31 participants had only 1 baseline visit datum available and thus, only 197 (71.1%) participants were included in the final analysis. These participants were equally distributed across the 3 study arms (lampalizumab 10 mg every 4 weeks, n = 63; lampalizumab 10 mg every 6 weeks, n = 68; pooled sham, n = 66). Only 1 eye per patient was selected as the study eye in the parent clinical trials and is reported herein.

Baseline characteristics were well distributed across the randomized treatment arms (Table 1). The majority of the population was White (98.5%). Fifty-two point eight percent of the total population was female and 47.2% was male. Mean age of patients across the study arms ranged from 75.9 to 78.4 years in this study, which was similar to the overall mean age in the Chroma and Spectri trials (78.0 years).^14^ Mean BCVA ranged from 66.9 to 68.6 ETDRS letters in this study, which was marginally better than that in the population from the Chroma and Spectri trials (66.0 letters).^14^ Baseline MP values are shown in Table 2.

**Table 1:** Pooled Demographics and Baseline Characteristics of Patients Enrolled in the Chroma and Spectri Clinical Trials and Included in the Present Analysis, by Treatment Group

Characteristics	LQ4W (n = 63)	LQ6W (n = 68)	Sham (n = 66)	Total (N = 197)
Demographics				
Sex				
Female	34 (54.0)	37 (54.4)	33 (50.0)	104 (52.8)
Male	29 (46.0)	31 (45.6)	33 (50.0)	93 (47.2)
Race, no. (%)				
White	62 (98.4)	67 (98.5)	65 (98.5)	194 (98.5)
American Indian or Alaska Native	0	1 (1.5)	0	1 (0.5)
Asian	1 (1.6)	0	0	1 (0.5)
Unknown	0	0	1 (1.5)	1 (0.5)
Age at parent reference date (years)				
Mean (SD)	75.9 (7.74)	78.4 (8.16)	77.8 (7.57)	77.4 (7.87)
Median (min, max)	76.0 (56.0, 90.0)	79.0 (58.0, 93.0)	78.5 (55.0, 94.0)	78.0 (55.0, 94.0)
Tobacco use, no. (%)				
Never	26 (41.3)	26 (38.2)	27 (40.9)	79 (40.1)
Previous	31 (49.2)	37 (54.4)	33 (50.0)	101 (51.3)
Current	6 (9.5)	5 (7.4)	6 (9.1)	17 (8.6)
Study eye baseline characteristics				
GA lesion area, mm^2^				
Mean (SD)	7.93 (4.26)	7.31 (3.81)	7.34 (3.77)	7.52 (3.93)
Median (min, max)	6.77 (2.56, 17.4)	6.32 (2.91, 17.7)	6.26 (2.56, 16.6)	6.34 (2.56, 17.7)
GA lesion contiguity, no. (%)				
Multifocal	53 (84.1)	55 (80.9)	55 (83.3)	163 (82.7)
Nonmultifocal	10 (15.9)	13 (19.1)	11 (16.7)	34 (17.3)
GA lesion location, no. (%)				
Subfoveal	32 (50.8)	37 (54.4)	31 (47.0)	100 (50.8)
Nonsubfoveal	31 (49.2)	31 (45.6)	35 (53.0)	97 (49.2)
GA lesion’s closest distance to foveal center point, μm				
Mean (SD)	124 (168)	99.0 (161)	172 (230)	131 (191)
Median (min, max)	0 (0, 539)	0 (0, 780)	85.0 (0, 957)	0 (0, 957)
Presence of reticular pseudodrusen, no. (%)				
No	34 (54.0)	36 (52.9)	41 (62.1)	111 (56.3)
Questionable	8 (12.7)	6 (8.8)	7 (10.6)	21 (10.7)
Yes	21 (33.3)	26 (38.2)	18 (27.3)	65 (33.0)
BCVA (ETDRS letters)				
Mean (SD)	68.1 (9.3)	66.9 (10.3)	68.6 (10.2)	67.9 (9.9)
Median (min, max)	68.0 (49.0, 89.0)	69.0 (50.0, 89.0)	70.0 (49.0, 90.0)	69.0 (49.0, 90.0)
Missing, no. (%)	0	1 (1.5)	1 (1.5)	2 (1.0)
Low-luminance BCVA (ETDRS letters)				
Mean (SD)	36.5 (19.2)	38.7 (14.8)	37.0 (17.1)	37.4 (17.0)
Median (min, max)	34.0 (7.00, 73.0)	38.0 (9.00, 65.0)	35.5 (4.00, 68.0)	35.0 (4.00, 73.0)
Missing, no. (%)	0	2 (2.9)	2 (3.0)	4 (2.0)
Low-luminance deficit (ETDRS letters)				
Mean (SD)	31.7 (16.6)	28.5 (13.7)	31.5 (17.3)	30.5 (15.9)
Median (min, max)	31.0 (4.00, 65.0)	27.5 (4.00, 63.0)	28.0 (2.00, 69.0)	29.0 (2.00, 69.0)
Missing, no. (%)	0	2 (2.9)	2 (3.0)	4 (2.0)

BCVA = best-corrected visual acuity; GA = geographic atrophy; LQ4W = lampalizumab 10 mg every 4 weeks; LQ6W = lampalizumab 10 mg every 6 weeks; max = maximum; min = minimum; SD = standard deviation.

**Table 2:** Baseline Microperimetry Values

Variable	LQ4W (n = 63)	LQ6W (n = 68)	Sham (n = 66)	Total (N = 197)
Mean macular sensitivity, dB				
Mean (SD)	5.94 (3.65)	6.52 (3.39)	6.48 (3.35)	6.32 (3.45)
Median (min, max)	5.29 (0.23, 15.4)	6.54 (0.03, 15.4)	6.81 (0.06, 12.3)	6.12 (0.03, 15.4)
Mean perilesional sensitivity, dB				
Mean (SD)	7.48 (2.79)	7.74 (2.80)	7.82 (2.72)	7.68 (2.76)
Median (min, max)	6.90 (3.27, 16.4)	7.28 (2.00, 15.4)	8.13 (2.00, 13.3)	7.33 (2.00, 16.4)
Mean responding sensitivity, dB				
Mean (SD)	8.92 (3.33)	9.17 (3.14)	9.16 (3.20)	9.09 (3.21)
Median (min, max)	8.33 (3.27, 17.1)	9.23 (2.00, 16.9)	9.74 (2.00, 14.6)	8.98 (2.00, 17.1)
Number of absolute scotomatous points				
Mean (SD)	26.2 (14.8)	22.9 (14.1)	23.4 (14.8)	24.1 (14.6)
Median (min, max)	25.0 (3.00, 66.0)	19.0 (4.00, 67.0)	17.5 (6.00, 66.0)	20.0 (3.00, 67.0)

LQ4W = lampalizumab 10 mg every 4 weeks; LQ6W = lampalizumab 10 mg every 6 weeks; max = maximum; min = minimum; SD = standard deviation.

### GA

**Figure 3:**
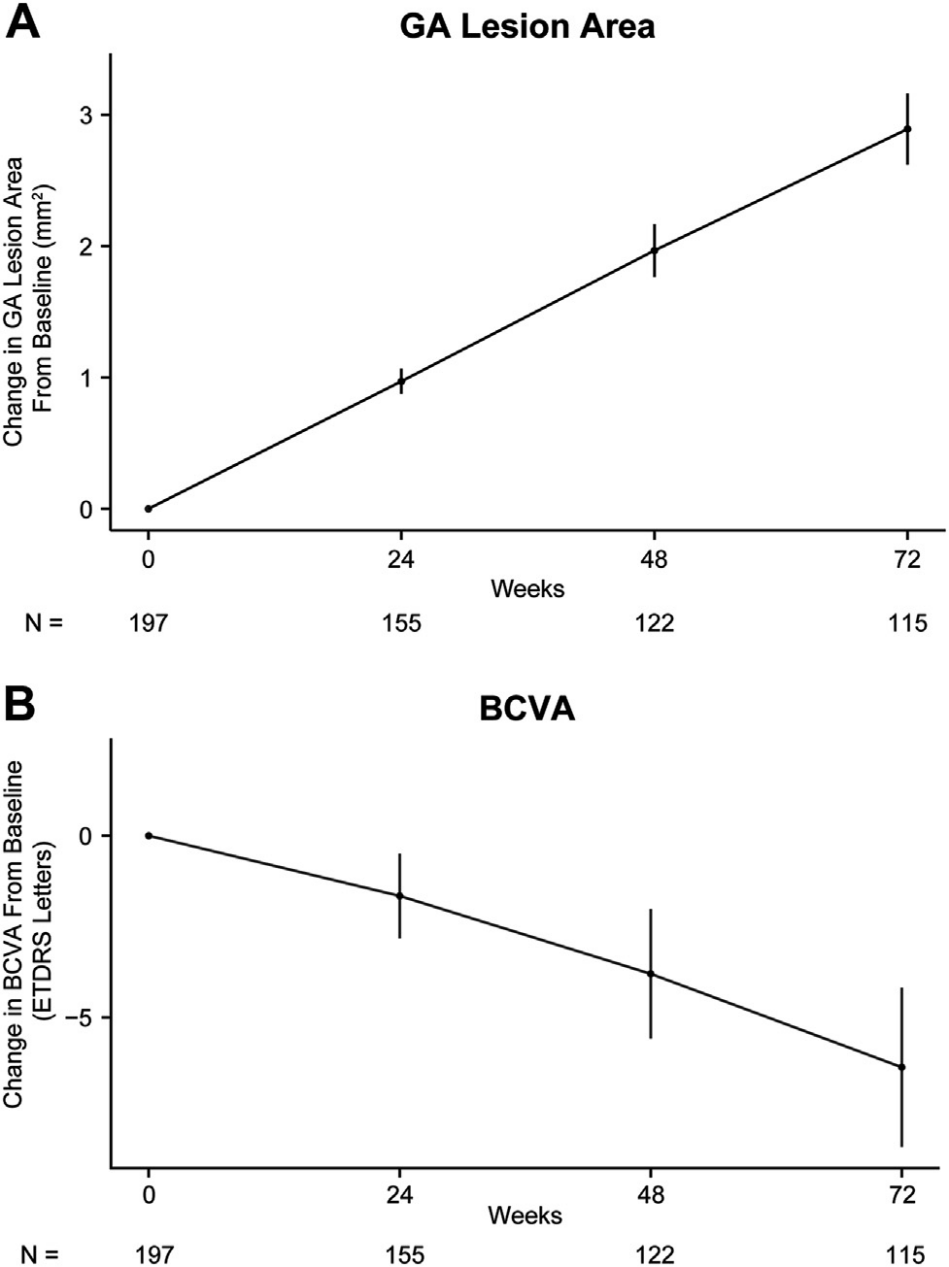
Change in (**A**) geographic atrophy (GA) lesion area and (**B**) best-corrected visual acuity (BCVA) from baseline to week 72. Error bars represent 2 times the standard error. N indicates the number of eyes per timepoint.

At week 48, mean change in GA lesion area in this study population was 2.0 mm^2^ (Fig 3A); the CV between all patients per visit was 56.5%. At week 72, mean change in GA lesion area increased to 2.9 mm^2^, whereas the CV decreased to 50.2%. Eye-level changes in GA lesion area over time are shown in Figure S4A (available at www.ophthalmologyscience.org).

### BCVA

Mean BCVA in this study population steadily declined over time (Fig 3B), with a mean decrease in BCVA of −3.8 letters at week 48 (CV = 253.4%) and −6.4 letters at week 72 (CV = 179.2%). Eye-level changes in BCVA over time showed high variability (Fig S4B).

### MP Endpoints

**Figure 5:**
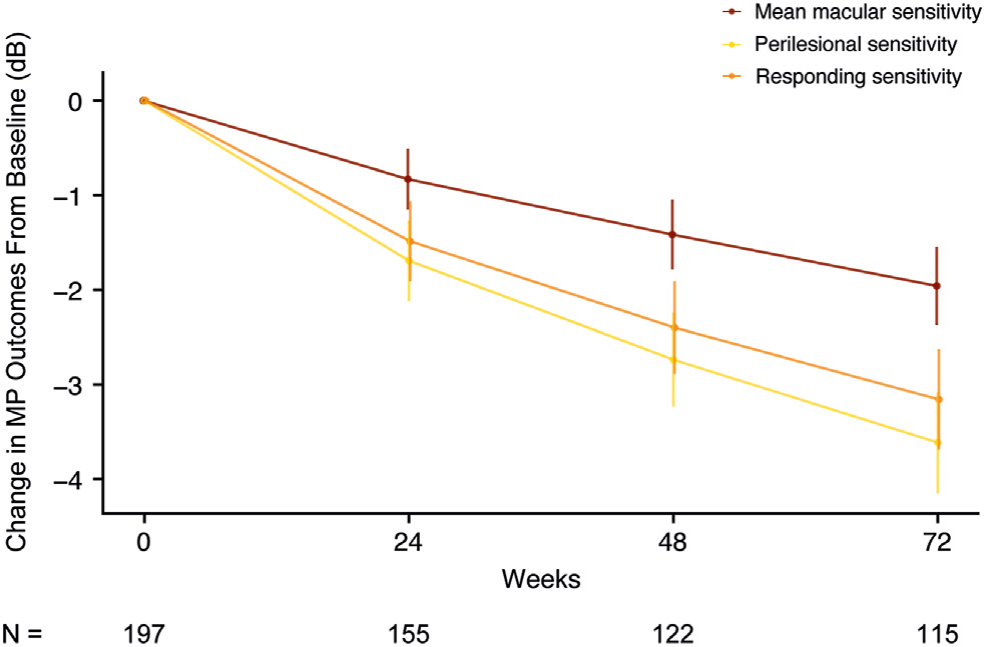
Longitudinal changes of different microperimetry (MP) outcomes from baseline to week 72. Error bars represent 2 times the standard error. N indicates the number of eyes per timepoint.

Macular sensitivity endpoints over time are shown in Figure 5. The commonly used mean macular sensitivity and number of absolute scotomatous points were compared with the proposed exploratory endpoints of perilesional and responding sensitivities. At week 48, mean change in mean macular sensitivity was −1.42 dB (CV = 144.01%) compared with −2.74 dB (CV = 100.38%) for perilesional sensitivity and −2.40 dB (CV = 112.04%) responding sensitivity (Table 3). At week 72, mean change in mean macular sensitivity was −1.96 dB (CV = 113.10%), compared with −3.61 dB (CV = 79.33%) for perilesional sensitivity and −3.16 dB (CV = 89.67%) for responding sensitivity. Mean change in the number of absolute scotomatous points at week 48 was 7.53 (CV = 127.65%) and at week 72 was 10.15 (CV = 106.74%; Table 3; Fig S6; available at www.ophthalmologyscience.org). Eye-level changes in MP endpoints over time demonstrated high levels of variability (Figs S4C to 4F).

**Table 3:** Microperimetry Endpoints

Endpoint	Mean Change (Coefficient of Variation, %)		
	*Week 24*	*Week 48*	*Week 72*
Mean macular sensitivity, dB	−0.83 (240.77)	−1.42 (144.01)	−1.96 (113.10)
Perilesional sensitivity, dB	−1.69 (155.94)	−2.74 (100.38)	−3.61 (79.33)
Responding sensitivity, dB	−1.49 (177.99)	−2.40 (112.04)	−3.16 (89.67)
Number of absolute scotomatous points	4.51 (188.33)	7.53 (127.65)	10.15 (106.74)

### Correlation between MP Endpoints and GA Lesion Area over Time

**Figure 7:**
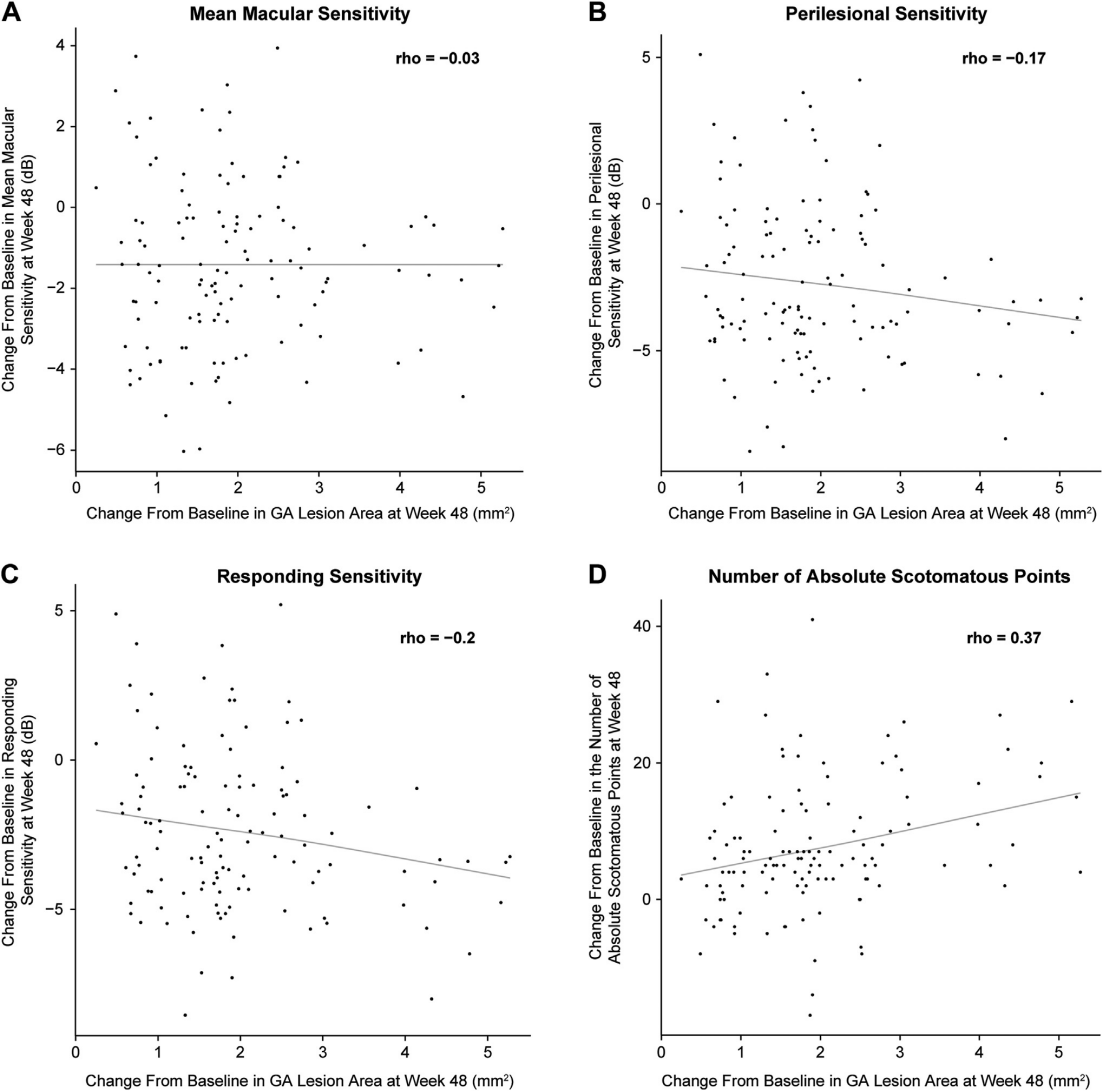
Association of geographic atrophy (GA) lesion area growth at week 48 with (**A**) mean macular sensitivity, (**B**) perilesional sensitivity, (**C**) responding sensitivity, and (**D**) number of absolute scotomatous points.

The correlation between MP endpoints and change in GA lesion area as determined by FAF at week 48 is shown in Figure 7. Mean macular sensitivity demonstrated the weakest correlation with GA lesion area (correlation coefficient: rho = −0.03). Perilesional and responding sensitivity had slightly improved correlation (rho = −0.17 and −0.20, respectively). The best correlation was observed between the number of absolute scotomatous points and GA lesion area (rho = 0.37).

## Discussion

In this exploratory study of the MP data from the Chroma and Spectri trials, we evaluated conventional as well as novel MP endpoints and determined their ability to detect visual function decline caused by GA secondary to AMD. We found that the mean GA lesion area enlarged by approximately 2 mm^2^/year, and all MP parameters deteriorated over time.

The novel MP endpoints of perilesional and responding sensitivities were more sensitive than the conventional MP endpoint of mean macular sensitivity for the measurement of functional decline over time. The lower magnitude observed in mean macular sensitivity is likely due to using all tested points, including absolute scotomatous points, which dilutes the mean. Perilesional and responding sensitivity exclude absolute scotomatous points identified at baseline and are therefore more sensitive than mean macular sensitivity, demonstrating higher change in dB with lower variability. The results of this study can be taken into consideration when planning the utilization of various MP testing patterns and when interpreting their outputs, including a standard grid for all patients, such as the one utilized in this clinical development program, as well as linear/cross or custom tailored patterns.

The assessment of the anatomic/functional correlation in GA has been challenging with many visual function tests. The correlation between GA lesion area over time and change in MP endpoints was the strongest for absolute scotomatous points. In alignment with previous studies,^10^^,^^13^ we confirmed that with the increase in GA lesion area, there was a resultant increase in absolute scotoma. However, the dichotomous nature of this endpoint means it cannot detect subtle reductions in retinal sensitivity and is therefore likely to be less sensitive in evaluating treatment effects than the perilesional and responding retinal sensitivity endpoints.

The correlation between GA lesion area and mean macular sensitivity was not as robust.^13^ This could be either because the parameter lacks spatial information and is blunted by the inclusion of absolute scotomatous points or because of the high variability observed in macular sensitivity measurements compared with GA lesion area.^13^ Perilesional and responding sensitivity correlated better than mean macular sensitivity, indicating that they might be potentially useful functional endpoints to consider in future studies.

Perilesional sensitivity captures the area adjacent to absolute scotoma.^13^ On FAF, this may correlate with abnormal hyperautofluorescence in some cases. It is unknown whether these areas are injured with the potential for recovery or if they are predestined to atrophy beyond the point of therapeutic recovery. If they are destined to atrophy, then responding sensitivity could potentially provide the best information on remaining macular response and visual function over time. If they can be rescued with a novel therapeutic intervention, then stable or improved function in the perilesional regions may be observed. Furthermore, categorizing these into perilesional and responding sensitivity could potentially provide some spatial resolution related to a hypothesized therapeutic effect.

Poor or eccentric fixation can impose additional challenges to the execution and interpretation of MP exams. Most MP examination strategies use real-time retinal tracking of a spatial map that may overcome some of the fixation issues common in patients with low vision.^17^^,^^18^ Using a retinal tracking map also improves test/retest variability compared with visual fields because it ensures the same macular region is retested at each visit.^17^ However, in this study, many patients had extensive foveal-involving GA lesions resulting in unstable or eccentric fixation that was difficult to overcome with retinal tracking alone.

Of note, to calculate the perilesional and responding sensitivities in this study, a mask of the MP map encompassing all absolute scotomatous points in the topography of the GA lesion area was created at baseline for each enrolled eye; this required the development of custom software (Fig 1). If similar sensitivity tests are to be performed in other studies aiming to validate treatment for GA, expert graders must be adequately trained and care must be taken when using these novel MP endpoints. Customized masks would be required for each patient.

The present study analyzes one of the largest MP datasets ever collected in macular diseases with prospectively enrolled patients evaluated at stringent intervals; notably, a standardized clinical trial–determined protocol of MP examination was followed, including rigorous data collection and analysis. In both of the clinical trials included in this study, > 98% of the population was White (Table 1). So, it is important to note that further considerations may be given in the future to ensure representation from various races and ethnicities in clinical trials of GA, in line with the demographic distribution of patients typically affected by the disease. The significance of this improvement has been previously noted by Baxter for retina clinical trials.^19^ One important limitation of this analysis is that the reproducibility or test/retest variability of the MP exam was not quantified in the Chroma and Spectri clinical trials and thus, the amount of variability expected in these endpoints of interest remains uncertain. In addition, it should be noted that the MP-1 microperimeter was used for this study; newer microperimeters, such as the iCare Macular Integrity Assessment microperimeter (CenterVue) or Nidek MP-3 microperimeter, that may provide better anatomic or functional correlation, are now available. A caveat of our study is that both perilesional and responding sensitivities as well as absolute scotomatous points (filled and zero points) were combined for statistical analysis. It is important to note that in clinical settings, the visual impairment represented by both groups is similar. In conclusion, we found MP to serve as a valuable visual function assessment in patients with GA. In addition to the conventional MP endpoints of mean macular sensitivity and number of absolute scotomatous points, results from this study allow us to propose perilesional and responding retinal sensitivity as novel MP endpoints that can capture visual functional decline over time with greater sensitivity. These functional endpoints may serve as a foundation for future clinical trials evaluating GA progression using MP, to better characterize both the natural history of the disease as well as early treatment response. Further research is warranted to evaluate the structural/functional correlation of the MP endpoints and to further optimize these by using newer parameters with improved registration, dynamic range, and efficient testing algorithms.
