# Supplementary material for: Macular Sensitivity Endpoints in Geographic Atrophy: Exploratory Analysis of Chroma and Spectri Clinical Trials
Source: Ophthalmol Sci. 2023 Jun 12;4(1):100351. doi: 10.1016/j.xops.2023.100351 (PMC10587617; doi:10.1016/j.xops.2023.100351)

Figure S4. Eye-level changes over time in (**A**) geographic atrophy (GA) lesion area, (**B**) best-corrected visual acuity (BCVA), (**C**) mean macular sensitivity, (**D**) perilesional sensitivity, (**E**) responding sensitivity, and (**F**) number of absolute scotomatous points. Individual eye-level changes over time are displayed to show how noisy the different endpoints are compared with changes in GA lesion area over time. The orange line indicates smoothing line (generalized additive model smoother) with formula y ~ s(x) using a shrinkage cubic spline basis with 4 knots. ETDRS = Early Treatment Diabetic Retinopathy Study

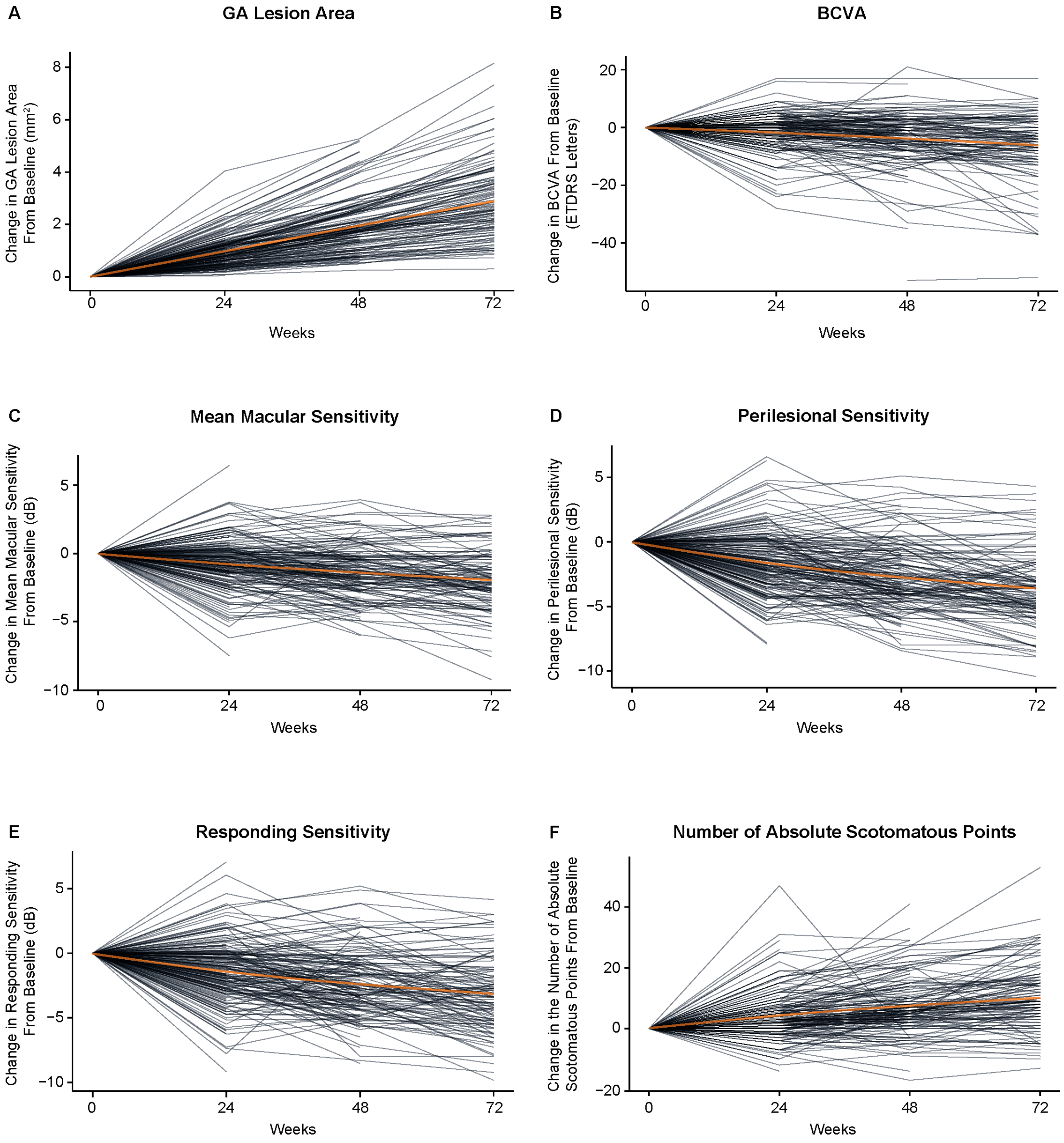

Supplement: Figure S4 [file mmc1.pdf]
