# Supplementary material for: Macular Sensitivity Endpoints in Geographic Atrophy: Exploratory Analysis of Chroma and Spectri Clinical Trials
Source: Ophthalmol Sci. 2023 Jun 12;4(1):100351. doi: 10.1016/j.xops.2023.100351 (PMC10587617; doi:10.1016/j.xops.2023.100351)

Figure S6. Mean change from baseline in the number of absolute scotomatous points. Error bars represent 2 times the standard error. N indicates the number of eyes per timepoint.

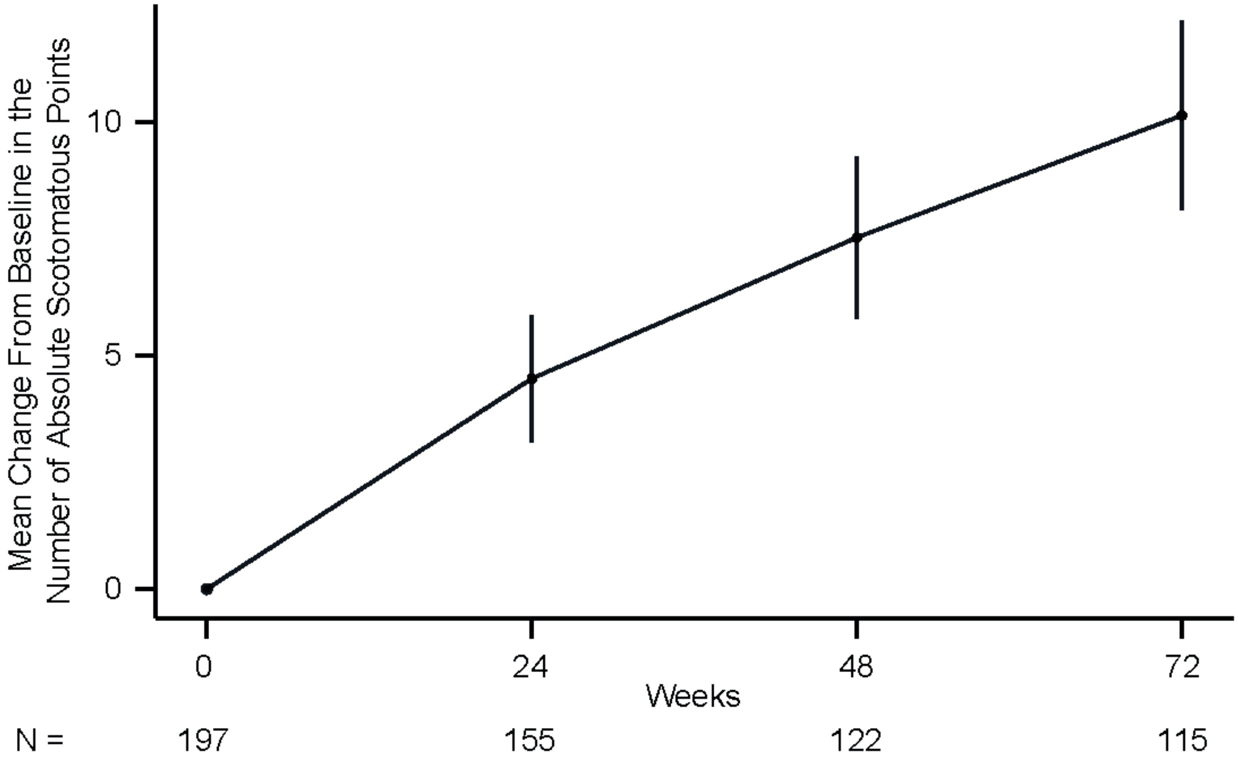

Supplement: Figure S6 [file mmc2.pdf]
